# Les luxations bipolaires de l'avant-bras (floating forearm)

**DOI:** 10.11604/pamj.2015.22.234.7890

**Published:** 2015-11-12

**Authors:** Badr Ennaciri, Mohamed Kharmaz

**Affiliations:** 1 Service de Chirurgie Orthopédique, CHU Avicenne, Rabat, Maroc

**Keywords:** Luxation du coude, luxation périlunaire, luxation radio-ulnaire distale, Elbow dislocation, perilunaire luxation, distal radioulnar dislocation

## Image en medicine

Les luxations concomitantes ipsilatérales du coude et du poignet (floating forearm) constituent des lésions très rares. Les luxations périlunaires surviennent suite à un traumatisme à haute énergie par hyperextension. Le diagnostic nécessite souvent le recours au scanner du poignet. Selon la force exercée sur le poignet, d'autres lésions peuvent y être associées. Le traitement est urgent et consiste en une réduction et stabilisation des articulations atteintes, suivi par un programme adapté de rééducation fonctionnelle du membre traumatisé. Nous rapportons l'observation d'un patient âgé de 56 ans, commerçant, droitier, tabagique chronique. Victime d'un accident de circulation avec traumatisme du membre supérieur gauche. L'examen clinique trouvait un avant-bras et main gauches œdématiés, coude et poignet déformés et ecchymotiques (A). L'examen vasculo-nerveux était normal. Les radiographies de l'avant-bras, du coude, du poignet et de la main gauches face et profil avec scanner du poignet, avaient objectivé des luxations postéro-externe du coude gauche, périlunaire antérieure du carpe et de la radio-ulnaire distale avec fracture diaphysaire de l'ulna, fracture de la styloïde radiale, fracture du scaphoïde carpien trans-tubérositaire type IV de Schernberg et fracture de la base du 1^er^ métacarpien (B,C). Le traitement avait consisté en une réduction orthopédique de la luxation du coude, ostéosynthèse de l'ulna par plaque spécial radius, double embrochage du scaphoïde, réduction et stabilisation de la luxation périlunaire et de la radio-ulnaire distale et embrochage de la base du 1^er^ métacarpien (D).

**Figure 1:**
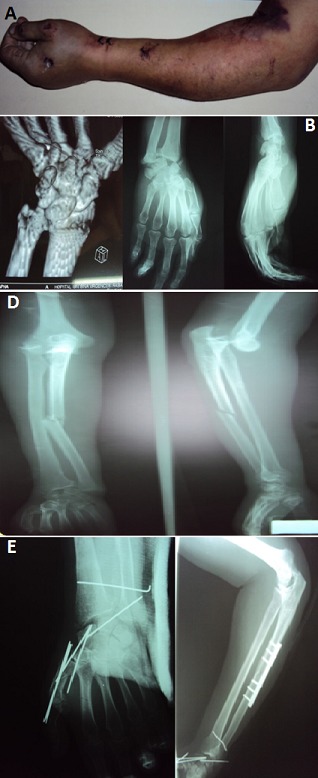
(A) avant-bras et main gauches œdématiés, coude et poignet déformés et ecchymotiques; (B) radiographie de la main gauche de face et de profil complétée par un scanner 3D confirmant la luxation périlunaire du carpe associée à la fracture transtubérositaire du scaphoïde type IV de Schernberg et à la fracture de la base du 1er métacarpien avec la déformation du poignet en Volarintercalated segmental instability; (C) radiographies de l'avant-bras gauche montrant les luxations postéro-externe du coude, de la radio-ulnaire distale et transcapho-périlunaire antérieure associées aux fractures de la diaphyse de l'ulna, de la styloïde radiale et de la base du 1er métacarpien; (D) radiographie de contrôle de l'avant-bras et de la main gauches montrant l'embrochage du scaphoïde, de la scapho-lunaire, de la radio-ulnaire distale et de la base du 1ermétacarpien et ostéosynthèse le l'ulna par plaque spécial radius après réduction de la luxation du coude

